# Sick at work: prevalence and determinants among healthcare workers, western Ethiopia: an institution based cross-sectional study

**DOI:** 10.1186/s40557-018-0213-4

**Published:** 2018-02-01

**Authors:** Tesfaye Hambisa Mekonnen, Mekuriaw Alemayewu Tefera, Yayehirad Alemu Melsew

**Affiliations:** 1 Department of Environmental and Occupational Health and Safety, Institute of Public Health, College of Medicine and Health Sciences, University of Gondar, P.O. Box 196, Gondar, Ethiopia; 2 Department of Epidemiology and Biostatistics, Institute of Public Health, College of Medicine and Health Sciences, University of Gondar, Gondar, Ethiopia

**Keywords:** Prevalence, Sickness Presenteeism, Health care workers, Cross-sectional, Ethiopia

## Abstract

**Background:**

Going to work despite feeling sick also known as sickness presenteeism is one of the emerging global occupational health challenges. Sickness presenteeism negatively affects both health of work forces and productivity of organizations in general. However, there is insufficient research exploring this situation in majority of the Sub-Saharan African countries, including Ethiopia. Thus this study intended to investigate the prevalence and determinant factors of sickness presenteeism among health care workers, Western Ethiopia.

**Methods:**

This study used an institution based cross-sectional quantitative study design. The study period was from February to March, 2017. We employed simple random sampling method to select 360 study samples. Data collection was performed by pre-tested structured and self- administered questionnaire. We used SPSS version 20 to carry out binary logistic regression analysis. Odds ratio with 95% confidence intervals was calculated and significance of associations was determined at *p*-value < 0.05.

**Results:**

A total of 344 respondents fully completed the survey questionnaire. Mean age with standard deviation was 30.28 ± 6.181. Prevalence of sickness Presenteeism was 52.6% [95%CI: (47.4, 57.8)] in the past 12 months. Educational status [AOR:2.1, 95%CI: (1.17,3.90)], financial problem [AOR:1.9,95%CI:(1.07,3.46)], sickness absenteeism [AOR:2.7,95% CI:(1.50,5.02)], lack of staff replacement [AOR:2.7,95%CI:(1.50,5.02)], absence of occupational health services [AOR:3.0,95%CI:(1.34,6.70)], and pressure from supervisor [AOR:1.8,95% CI:(1.01,3.31)] were significant predictors of the dependent variable.

**Conclusion:**

Relatively higher proportions of workers indicated sickness presenteeism as compared to other studies. Risk factors like educational status, personal financial problem, sickness absenteeism, lack of staff replacement, absence of occupational health services, and pressure from supervisors considerably increased the likely occurrence of employees’ sick attendance. It is advisable for health care managers to hire adequate health care staffs, to implement basic occupational health services and to design strategies which reduce pressure from supervisors.

## Background

Going to work despite feeling sick also known as sickness presenteeism is one of the emerging global occupational health challenges. Sickness presenteeism negatively affects both health of work forces and productivity of organizations in general. It is the concept where workers decide to go to work while they are being distracted and not at their full performance capacities [1, 2]. In this study, the term sickness presenteeism is interchangeably used with the terms sick attendance, ill presence, going to work despite feeling sick, and sick at work. Moreover, for the purpose of this study, all these terms reflect the same central impression in connotation.

Working in spite of illness can lead to many negative consequences both to the workers and to the organizations in terms of productivity loss (due to lost productive time), poor quality (while workers make mistakes), impaired social functioning, low workers’ morale (bad images and loss of confidence while making errors) and job insecurity/turnover (it may negatively affect employers–employees relationship [3–5]. Sickness presenteeism can exacerbate the existing ill health due to restricted opportunities for workers to take a rest and this may cause workers to require prolonged recovery from particular health conditions [6, 7]. Evidences in the literature also demonstrated that working while sick can lead to work disability at a later date [4, 5, 8]. Moreover, studies explored that working despite sickness is a risk factor for many negative health outcomes to employees. For example, depression, burnout, and serious cardiac events are commonly reported negative health outcomes among healthcare providers [9]. Further, healthcare workers who attend work with contagious illnesses can be possible sources for public health problems in terms of diseases transmission and outbreak extensions [6, 10–12].

Literatures revealed that workers’ ill presence is prominent among different working groups. For instance, sickness presenteeism was indicated in about 48.7% of participants in New Zealand hospital [11]. A third of 3801 participants of one study [13] and 53% of another study [6] in Sweden had reported that they had gone to work while they were feeling sick. Another study reported 56% prevalence of sickness presenteeism among Norwegian and Swedish respondents in the past 12 months [14]. A comparative cross-sectional study findings among physicians of four European countries (Sweden, Norway, Iceland and Italy) also reported 70%–86% prevalence of employees’ ill presence [15].

Different theoretical framework models in the literature also explored the association between socio-demographic characteristics like age, sex, education, profession [6, 16, 17], personal factors such as financial pressure, sickness absenteeism, boundarylessness [6, 13, 18], and work related factors like lack of staff replacement, sick pay, attendance policy [6, 13] and sickness presenteeism. Furthermore, earlier studies also investigated the positive relations of health conditions such as musculoskeletal disorders including low back pain, diabetes, and migraine [17, 19, 20] and behavioral factors such as physical inactivity, smoking and alcohol consumptions [21].

Even though sickness presenteeism is common in all working populations, studies have shown that its prevalence increases in the care and welfare, and education sectors [13]. Health care workers are more likely at risk than the other work forces because the service they provide is customer contact in its nature. This could in turn compel to the high physical presence requirement for such categories of workers. Healthcare workers’ attending work while sick not only puts patients at risk [7] but also decreases productivity and increases the probability of medical errors [9]. Hence, in healthcare sectors, the contemporary growing concerns for quality healthcare including the need for medical efficacy and patient safety outcomes and the rising costs of health care (such as costs due to employees’ health protection programs) are few of the motives for studying sickness presenteeism as an important research agenda worldwide [1, 22].

However, there is a dearth of evidence on the magnitude and range of factors giving rise to sickness presenteeism among health care employees in majority of the Sub-Saharan Africa, including Ethiopia. The present study was intended to explore the extent and factors associated with sickness presenteeism among health care providers in Western Ethiopia. The findings of the study would likely contribute solid and valuable information for policy makers (particularly health care managers) and other stake holders to devise the required preventive approaches.

## Methods

### Study design

An institution based cross-sectional quantitative study.

### Study area and period

The study was carried out in East and West Wollega Zones public hospitals, Western Ethiopia, from February to March, 2017. The two zones are nearby zones and located at about 320 and 495 km from Addis Ababa, the capital of Ethiopia, respectively. There are 4 public hospitals in the two Zones employing about more than 900 healthcare providers. The 4 hospitals were purposively included to attain the required sample size.

### Source population

All healthcare workers who have been working in East and West Wollega Zones public hospitals were the source population of the study.

### Inclusion and exclusion criteria

### Inclusion criteria

All clinical staff workers including doctors (medical and dentists), nurses, midwives, pharmacists, laboratory technologists, anesthesia, psychiatrists, physiotherapy, optometry, radiologists and health officers) who have been working for at least 12 months prior to the study period were included.

#### Exclusion criteria

Administrative and supportive staff workers were excluded. Clinical staff workers who were on annual and sick leave and women health care workers who were on maternity leave were also excluded.

### Sample size and sampling procedure

Epi info version 7 was used to determine the sample size required for this study with 50% expected proportion, 4% confidence limit, and 95% confidence level. By considering 10% non-response rate, the final sample size was 360. Simple random sampling method was used to select the proposed samples. Workers were then proportionally allocated according to the number of workers in each hospital. Finally, Open Epi (computer generated random number) was employed to obtain the participants.

### Data collection tools and techniques

Data were collected using pre-tested structured and self-administered questionnaires. All the structured questionnaires were prepared from literature with a slight modification [1, 10, 21, 23]. A Standardized Nordic Musculoskeletal questionnaire was employed to assess Musculoskeletal disorders [24]. The questionnaire was divided in to four parts. The first part was socio-demographic characteristics like gender, age, educational status, profession, and marital status. The second section included personal factors such as financial problem, sickness absenteeism and workplace factors like lack of replacement availability, absence of occupational health service, pressure from team leader/supervisor, and the third and fourth part were behavioral characteristics including cigarette smoking and physical activity and specific health conditions (Diseases and injuries) respectively.

### Data quality control

First, we gave high emphasis for the data collection tools. The assessment tool was first developed in English and then translated to local language (Afaan Oromiffa) and back to English by language experts for its consistency. Second, five data collectors and three supervisors were trained for 2 days concerning data collection tools, data collection time, exclusion and inclusion criterion and ethical issues. Third, we conducted pre-test on 21 workers of the other nearby zone public hospital which has characteristics nearly similar to the participants under study to identify the possible misinterpretations, and other related objections that may happen to any of the survey tool. Based on the pre-test results, the number of questionnaire was reduced and some misinterpretations were modified. Finally, we checked the data for completeness before entry and cleaned before analysis.

### Study variables

Sickness presenteeism was a dependent variable of the study. It was measured by using a single item: In the previous 12 months, have you gone to work while feeling sick, even though it would have been really reasonable to take sick leave? The four point response scale was (1.No, never; 2.Yes, once; 3.Yes, 2–5 times; 4. Yes, more than 5 times). For the purpose of logistic regression analysis, the response scale was dichotomized in to (0 = No, never/Yes, once; 1 = Yes, ≥2 times). Workers who indicated that they had gone to work despite their sickness in past 12 months two times and more were considered as experiencing sickness presenteeism. This item was frequently used by other previous studies in the literature [6, 8, 13, 25–27].

A self-reported weight and height of the respondents was used to calculate body mass index as weight divided by squared height (Kg/M^2^). The respondents were also questioned to indicate their monthly salary in Ethiopian Birr (ETB). Work experience was obtained in years. The lists of specific health conditions most frequently diagnosed in the area were provided to select from the lists if they might have been experiencing the condition/conditions in the past 12 months. They were also asked to indicate any other particular diseases and injuries if their option/options was/were not in the lists provided. Finally, all the self-report health conditions were indicated by Yes/No response. Sickness absenteeism was measured by the number of sick leave used due to illness twice and more in the previous 12 months. All the remaining variables were measured by Yes/No response scale.

### Data analysis

We employed Epi-Info version 7 to enter and clean data. Statistical Package for Social Sciences (SPSS) version 20 soft ware was used for data analysis. Results were described using tables, graphs and summarized by percentages, means and standard deviations. Collinearity test was checked using Variance Inflation Factor (VIF) and the assumption was fulfilled (VIF < 10). Independent variables were fitted separately in to bivariate logistic regression analysis to examine the degree of association with sickness presenteeism. Variables with a *p*-value of < 0.2 in bivariate analysis were exported to multivariate logistic regression model to control the possible confounders. A marginal point of p-value < 0.2 was also assumed because more study variables (above 40) were evaluated in this study.

A backward variable selection method was used in multivariate logistic model. This was employed because it is assumed to be relatively more conservative type of variable selection methods. Goodness of fit for model was checked by Hosmer and Lemeshow test and the assumption was also satisfied (*p* > 0.05). Significance of association was obtained at odds ratios with 95% confidence intervals and *p*-value cut off < 0.05 was considered as a statistically significant association.

## Results

### Socio-demographic characteristics

Response rate was 95.56% (*N* = 344). Majority of the participants 201 (58.4%) were males. Age ranges from 22 to 55 years with a mean and standard deviation of 30.28 ± 6.181. Of the participants, 165 (48%) of them were first degree and 131 (38%) diploma and below holders (Table 1).

**Table 1:** Characteristics of HCW in relation to SP, Western Ethiopia, 2017 (*N* = 344)

Variables	Frequency	Percentage (%)
Sex		
Female	143	41.6
Male	201	58.4
Age group		
18–30	229	66.6
31–44	97	28.2
45–60	18	5.2
Marital status		
Unmarried/single	124	36.0
Married/Cohabiting	210	61.0
Widowed /separated/divorced	10	3.0
Professional category		
Nurses	124	36.0
Midwives	48	14.0
Doctors	37	11.0
Laboratory technologists	28	8.0
Pharmacists	28	8.0
Others professionals^a^	79	23.0
Educational status		
Diploma and below	131	38.0
First degree	165	48.0
Doctorate (medical and dentists)	37	10.8
Masters (clinical)	11	3.2
Work experience		
1–5 years	133	38.7
6–15 years	175	50.9
> 15 years	36	10.5
Type of employment		
Temporary	7	2.0
Permanent	337	98.0

Keys: - ^a^ = Anesthesia, Health officers, Optometrists, Physiotherapists, Psychiatrists, Radiologists, HCWs = Healthcare workers; *N*=Number; SP=Sickness Presenteeism

### Personal and work related factors

Regarding personal related characteristics, 157 (45.6%) of the respondents indicated that they attended work despite their sickness to get duty care payment and other benefits/for personal financial problem/ and 180 (52.3%) of the respondents attended work despite their illness because they find it hard to say no/individual boundarylessness.

**Fig. 1:**
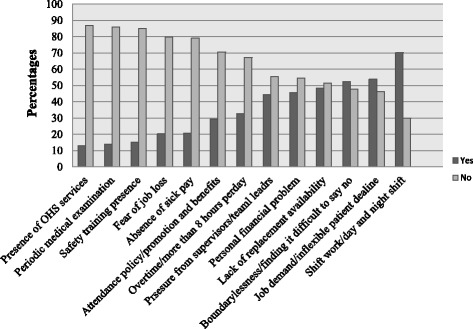
Personal and work related characteristics of health care workers, Western Ethiopia, 2017 (*N* = 344). Keys: - Not applicable

Work related factors were also the main reasons of sickness presenteeism. Out of the total participants, 177 (51.1%) of them attended work in spite of their sickness due to lack of replacement availability/staff shortage and 153 (44.5%) reported that they attended work despite their health conditions due to pressure from supervisors/team leaders (Fig. 1).

### Prevalence and frequencies of sickness Presenteeism

The overall prevalence of sickness presenteeism was 52.6% [95%CI: (47.4, 57.8)] during the previous 12 months. Majority, 105 (30.5%) of them were male health care workers. Sickness presenteeism was indicated high, 114 (33.1%) among 18–30 age group. Higher prevalence of sick attendance, 125 (36.3%) showed to be 2–5 times whereas 56 (16.3%) of the respondents experienced greater than 5 times **(**Table 2**).**

**Table 2:** Characteristics of HCWs in relation to frequency of SP, Western Ethiopia, 2017 (*N* = 344)

Variables	No (%)	SP in the Past 12 months			
		No, never	yes, once	yes, 2–5 times	yes, > 5 times
Sex					
Female	143(41.6)	27	40	60	16
Male	201(58.4)	54	42	65	40
Age					
18–30	229(66.6)	61	54	86	28
31–44	97(28.2)	19	24	33	21
45–60	18 (5.2)	1	4	6	7
Marital status					
Unmarried/single	124(36)	37	32	40	15
Married/Cohabiting	210(61)	43	46	82	39
Widowed /separated/divorced	10(3)	1	4	3	2
Educational status					
Diploma and below	131(38.31)	30	42	43	16
First degree	165(48)	43	35	67	20
Doctorate	37(10.8)	7	4	11	15
Masters	11(3.2)	1	1	4	5
Profession					
Doctors	37(10.8)	5	6	15	11
Nurses	125(36.3)	27	27	49	22
Midwives	49 (14.2)	14	12	17	6
Pharmacists	26 (7.6)	6	9	9	32
Laboratory technologists	29(8.4)	5	10	11	3
Other professionals^a^	78(22.7)	24	18	24	12
Work experiences					
≤ 5 years	133(38.7)	39	36	42	16
6–15 years	175(50.9)	38	37	71	29
> 15 years	36(10.5)	4	9	12	11
Type of employment					
Temporary	7(2)	1	2	2	2
Permanent	337(98)	80	80	123	54

**Keys**: ^a^ = Radiologists, Anesthesia, Optometry, Health officers, Physiotherapy, Psychiatrists; HCW = Health care workers; SP=Sickness Presenteeism; No = Number; % = Percentage

### Indicated causes of sickness Presenteeism

**Fig. 2:**
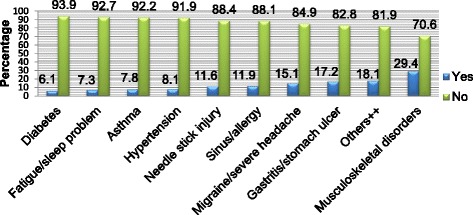
The indicated causes of Sickness Presenteeism among health care workers, Western Ethiopia, 2017 (*N* = 344). Keys: ++ = Hepatitis, Cardiac problem, Respiratory infection, Dermatitis, Hemorrhoids, Influenza, Pneumonia, Arthritis, Tuberculosis, Typhoid, Malaria, Gastro enteritis

Experiencing musculoskeletal disorders, 101 (29.4%) was the major reason for workers ill presence. Hypertension, 28 (8.1%) and Diabetes, 21 (6.1%) were also common health conditions leading to employees’ sickness presenteeism and these health problems were considered as chronic health conditions (participants had experienced for 3 months and more). Typhoid, 36 (10.5%), 24 (7%) malaria, and 44 (12.8%) gastroenteritis were also the other disease conditions causing workers’ ill presence and these were categorized as the self-reported acute disease conditions. Moreover, experiencing more than one disease conditions (co morbidities) was also observed to be the reason for 35 (10.2%) of the respondents sick attendance (Fig. 2).

### Factors associated with sickness Presenteeism

Of the predictor variables included in the multivariate logistic regression model, educational status, financial problem, sickness absenteeism, staff replacement, absence of occupational health services, and pressure from supervisor were investigated to be significant predictors of employees’ ill presence.

Participants of first degree and above holders in educational status were 2.13 times more likely to experience sickness presenteeism than participants of diploma and below holders [AOR: 2.13, 95% CI: (1.16, 3.89)]. Respondents who indicated having personal financial problem were 1.93 times at risk for sick attendance [AOR: 1.93, 95%CI: (1.07, 3.45)] than those who did not indicate. The likely hood of workers’ ill presence increased among participants who repeatedly used sick leave/sickness absenteeism [AOR: 2.74, 95% CI: (1.50, 5.02)] than those who didn’t use. Multivariate logistic regression model showed that the odds of sickness presenteeism was 2.64 times higher among respondents who reported lack of staff replacement availability [AOR: 2.64, 95% CI: (1.46, 4.78)] than those who didn’t report. Absence of occupational health services at the hospitals also 2.99 times more likely increased the occurrences of sickness presenteeism [AOR: 2.99, 95% CI:(1.34,6.69)]. Moreover, sickness presenteeism was indicated 1.83 times more likely due to pressure from supervisor [AOR: 1.83, 95%CI: (1.01, 3.31)] at the hospitals than where there is no pressure from supervisors. In this study, however, age, sex, profession, health conditions/acute and chronic/, comorbidities, shift work, and behavioral factors didn’t show any significant relation with dependent variable of the study (Table 3).

**Table 3:** Factors associated with SP among health care workers, Western Ethiopia, 2017 (*N* = 344)

Variables	Sickness presenteeism		COR (95% CI)	AOR (95% CI)	*p-values*
	Yes	No			
Marital status					
Unmarried/single	55	69	1.00	1.00	
Married/Cohabiting	121	89	1.71 (1.09–2.669)	1.63 (0.86–3.09)	p = 0.019*
Widowed /separated/divorced	5	5	1.26 (0.34–4.55)	0.88 (0.15–5.15)	
Educational status					
Diploma and below	59	72	1.00	1.00	
First degree and above	122	91	1.64(1.05–2.53)	2.13(1.166–3.89)	p = 0.014**
Profession					
Doctors	26	11	2.76 (1.19–6.34)	1.84 (0.57–5.90)	p = 0.017*
Nurses	71	54	1.53 (0.86–2.70)	1.88 (0.84–4.20)	
Midwives	23	26	1.03 (0.50–2.11)	0.90 (0.35–2.28)	
Pharmacists	11	15	0.86 (0.34–2.09)	1.31 (0.37–4.61)	
Laboratory technologists	14	15	1.09 (0.46–2.55)	0.89 (0.25–3.18)	
Other professionals^a^	36	42	1.00	1.00	
Personal financial problem					
No	86	101	1.00	1.00	
Yes	95	62	1.8(1.17–2.767)	1.93 (1.07–3.45)	p = 0.028**
Health problems/conditions					
Acute	76	72	1.00	1.00	
Chronic	105	37	2.68 (1.64–4.40)	0.85 (`0.39–1.85)	p = 0.0001*
sickness absenteeism/Sick leave used					
Never take sick leave	96	120	1.00	1.00	
Sick leave used twice and more	85	43	2.47 (1.56–3.89)	2.74 (1.49–5.02)	p = 0.001**
Lack of replacement availability					
No	76	101	1.00	1.00	
Yes	105	62	2.25 (1.46–3.46)	2.64 (1.46–4.78)	p = 0.001**
Presence of basic occupational health service at hospitals					
No	162	137	1.61 (0.85–3.05)	2.99 (1.34–6.69)	p = 0.007**
Yes	19	26	1.00	1.00	
Pressure from teams leader/supervisors					
No	89	102	1.00	1.00	
Yes	92	61	1.72 (1.12–2.65)	1.83 (1.01–3.31)	p = 0.047**

Keys: - 1.00 = Reference category; other^a^ = Anesthetists, Health Officers, Optometrists, Physiotherapists, Psychiatrists, Radiologists;* = significant in bivariate logistic regression model;** = significant in multivariate logistic regression model; AOR = Adjusted Odds ratios; CI=Confidence Interval; COR = crude Odds ratios

## Discussion

Sickness presenteeism is the emerging occupational health challenges facing health care providers in developing countries including Ethiopia, with scant research attentions. This study primarily intended to explore the magnitude and risk factors associated with sickness presenteeism among healthcare workers in public hospitals. The overall prevalence of sickness presenteeism in present study was found to be 52.6% (*N* = 181) during the past 12 months.

This magnitude was relatively comparable with the study conducted in New Zealand hospitals 48.7% [11], study from Belgium 50.6% [28] and 57% prevalence reported from other country [29]. Providing common services such as treating patients in general and mandatory workers physical presence during surgical procedures for instance might be possible reasons. However, this finding was slight lower than a comparative study conducted across four countries of Italia (Prevalence 86%), Sweden (70%), Norway (76%) and Iceland (75%) [15] and study from Brazil (75%) [30]. This difference might be explained by discrepancies in characteristics of the population under the studies. Other possible suggestion may be due to that the degree to which illness might be perceived among participants. Those participants of countries with a relatively good economic status might perceive very less severe illness as a major concerned health problem but those with poor economic status counterparts might consider those similar conditions even not as illness and therefore choose to attend work despite their conditions. More possible explanation might be due to the issues of confidentiality and privacy discrepancies of participants among the countries. Countries which reported relatively high prevalence might address confidentiality and privacy issues of illness at work for instance, workplace arrangement for this purpose might be there and might not be a subject but this might be an issue in current study.

The level of education of participants in this study was significantly associated with sickness presenteeism. This result was consistence with the findings of another studies [14, 31]. Possible suggestion might be due that relatively more educated workers (in this study, first degree and above holders) might have high job attendance requirements. Another possible reason might be attributed to the ability to control over work tasks. Relatively more educated workers might be more likely to have high degrees of control over their work tasks under dreadful situations persisting in to go to work in spite of their sickness. More possible explanation might also be due that there might be extra responsibility assignments for a relatively more educated participants, as was also suggested by other studies [6, 14].

Multivariate logistic regression analysis had also revealed a significant association between workers’ ill presence and lack of staff replacement availability due to under staffing. This was in line with two independent studies reported from Sweden [6, 13] and other study from Canada [1]. Possible explanation might be due to staff scarcity and lack of multi skilled professionals, nobody might not cover the work of others apart from the assigned persons for that specific job activities and fear of job burden such as more working hours on return from sick to work might be there. Other explanatory suggestion might be due to availabilities of a very few highly specialized health care providers in Ethiopia, there might be a strict policy ground for such workers with regard to their absence.

Personal financial problem was also identified as a significant predictor of sickness presenteeism. Study from Sweden [6] and two independent studies from the USA [31, 32] had also reported similar findings. Participants who have financial problems might lack other opportunity to cover their financial problems if reduction from their monthly wages might be the rule and regulation of their particular work place because of repeated sickness related absence from work. Other alternative explanation could be due to existing policies and regulations on incentives and other extra benefits for works done beyond normal working hours and other categories of works such as duty cares that generate such payments, and which workers might be unwilling to miss them due to their financial problems.

This study indicated a positive association between absence of basic occupational health services and sickness presenteeism. There is lack of research exploring the significant association between occupational health services and sickness presenteeism. It is therefore difficult to interpret this finding. The probable reason for the significant association however, might be due to the more likely exposure to different hospital environmental hazards because of the existing poor implementation of occupational health services and poor attention of workplace illness management in Ethiopia. Provision of basic occupational health services could improve the work environment which in turn promotes workers’ health and safety. Furthermore, properly implemented work place health and safety possibly enhance workers awareness and prevention of at work illnesses. Work place injury and illness management are important components of occupational health services. Study from Korea reported the significant association of sickness presenteeism and work place environmental risk exposure [16]. The authors would like to suggest the researchers to further verify the association of occupational health services and workers sick attendance.

Pressure from team leader/supervisor indicated to be significant predictor. Consistence with study conducted in Australia [23] and studies from USA [31, 33], team leader/supervisor pressure increased workers ill presence. Workers who are influenced by a negative supervisor behavior probably become stressed and could find it hard to decide to take a rest. The other possible reason might also be due to team leaders/supervisors’ lack of understanding about the potential negative outcomes associated with working while sick, so that some team leaders/supervisors may give value for workers’ physical presence alone, regardless of their ability to accomplish their duties.

The finding of this study also demonstrated statistically significant relation of sickness absenteeism and sickness presenteeism. Studies from Belgium [34], Canada [1] and Sweden [13, 35] also reported similar findings. Explanation is that frequently inappropriate use of sick leave/sickness absence itself might negatively affect employment relation of the employees, leading them to stay at work while they were sick. Workers might also already have finished their legitimate sick leave and use of extra sick leave might be difficult, which again may increase the likely occurrences of employees’ ill presence.

This study may meet with some limitations. First, the information obtained on sickness presenteeism was based on self-report of the participants. Therefore, the problem of recall bias and under reporting of cases might be suspected. However, to decrease recall bias, lists of health conditions usually diagnosed in the area were provided to check all that pertinent. Furthermore, we assessed health conditions related to work characteristics such as musculoskeletal disorders by using independent tools. Second, the sample did not include administrative and supportive staffs but the problem may also affect them alike. Therefore, generalizing for all working group might be the other study’s drawback.

## Conclusion

Relatively higher proportions of workers indicated sickness presenteeism as compared to the other studies. Risk factors like educational status, personal financial problem, sickness absenteeism, lack of staff replacement, absence of occupational health services, and pressure from supervisors considerably increased the likely occurrence of employees’ sickness presenteeism. It is advisable for health care managers to hire adequate health care staffs, to implement basic occupational health services and to design strategies which reduce pressure from supervisors.

## Abbreviations

AOR: Adjusted Odds Ratio
CI: Confidence Interval
COR: Crude Odds Ratio
MPH: Master of Public Health
PhD: Doctor of philosophy
SP: Sickness Presenteeism
SPSS: Statistical Package for Social Sciences
USA: the United States of America
